# Estimation of Effect of Radiation Dose Reduction for Internal Exposure by Food Regulations under the Current Criteria for Radionuclides in Foodstuff in Japan Using Monitoring Results

**DOI:** 10.3390/foods10040691

**Published:** 2021-03-24

**Authors:** Minoru Osanai, Daisuke Hirano, Shiori Mitsuhashi, Kohsei Kudo, Shota Hosokawa, Megumi Tsushima, Kazuki Iwaoka, Ichiro Yamaguchi, Takakiyo Tsujiguchi, Masahiro Hosoda, Yoichiro Hosokawa, Yoko Saito

**Affiliations:** 1 Department of Radiation Science, Hirosaki University Graduate School of Health Sciences, Hirosaki, Aomori 036-8564, Japan; kohsei@hirosaki-u.ac.jp (K.K.); shosokawa@hirosaki-u.ac.jp (S.H.); tmegumi@hirosaki-u.ac.jp (M.T.); r.tsuji@hirosaki-u.ac.jp (T.T.); m_hosoda@hirosaki-u.ac.jp (M.H.); hosokawa@hirosaki-u.ac.jp (Y.H.); yokosait@hirosaki-u.ac.jp (Y.S.); 2 Department of Radiological Technology, Hirosaki University School of Health Sciences, Hirosaki, Aomori 036-8564, Japan; h17m2231@hirosaki-u.ac.jp (D.H.); h17m2236@hirosaki-u.ac.jp (S.M.); 3 Center for Radiation Protection Knowledge, National Institute of Radiological Sciences, National Institutes for Quantum and Radiological Sciences and Technology, Inage, Chiba 263-8555, Japan; iwaoka.kazuki@qst.go.jp; 4 Department of Environmental Health, National Institute of Public Health, Wako, Saitama 351-0197, Japan; yamaguchi.i.aa@niph.go.jp

**Keywords:** radionuclides, standard limits, food monitoring test, Fukushima Dai-ichi Nuclear Power Plant accident, food regulation, food safety, internal exposure dose, risk assessment

## Abstract

This study examined the effect of food regulations under the current criteria (e.g., 100 Bq/kg for general foods) established approximately a year after the Fukushima Dai-ichi Nuclear Power Plant (FDNPP) accident. Foods are monitored to ensure that foods exceeding the standard limit are not distributed; ~300,000 examinations per year have been performed especially since FY2014. This study comprehensively estimated the internal exposure dose resulting from the ingestion of foods containing radioactive cesium using the accumulated monitoring results. Committed effective dose was conservatively calculated as the product of the radioactive concentration randomly sampled from test results, food intake, and dose coefficient. The median, 95th, and 99th percentile of the dose were 0.0479, 0.207, and 10.6 mSv/y, respectively, in the estimation with all test results (without regulation), and 0.0430, 0.0790, and 0.233 mSv/y, respectively, in the estimation with results within the standard limits (with regulation) in FY2012. In FY2016, the dose with and without regulation were similar, except for high percentile, and those doses were significantly smaller than 1 mSv/y, which was adopted as the basis for the current criteria. The food regulation measures implemented in Japan after the FDNPP accident have been beneficial, and food safety against radionuclides has been ensured.

## 1. Introduction

The Fukushima Dai-ichi Nuclear Power Plant (FDNPP) accident was caused by the 11 March 2011 Great East Japan Earthquake and tsunami. Due to the hydrogen explosions at the FDNPP, many types of radionuclides were released into the environment [1]. The Ministry of Health, Labour, and Welfare (MHLW) in Japan set the provisional regulation values for radionuclide in foods on March 17 as an urgent response [2,3,4,5]. The provisional regulation values were established as radionuclide concentration based on an effective dose of 5 mSv/y for radioactive cesium. For instance, the value for radioactive cesium in drinking water and milk/dairy products was 200 Bq/kg, and that in vegetables, grains, meat, eggs, and fish was 500 Bq/kg. These values of radioactivity concentration were set including the contribution of radioactive strontium to the radiation dose. In addition, the provisional regulation values were also set for isotopes such as radioactive iodine based on a scenario different from that of radioactive cesium. New standard limits (i.e., the current criteria), which were intended for the existing exposure situation, was established on 1 April 2012 based on the discussion in the Pharmaceutical Affairs and Food Sanitation Council [6]. The current criteria as radionuclide concentration for radioactive cesium (sum of cesium-134 (Cs-134) and cesium-137 (Cs-137)) was calculated based on 1 mSv/y. The current criteria are listed in Table 1. The value of 1 mSv/y is consistent with a reference level adopted by the Codex Alimentarius Commission (CAC) based on the International Commission on Radiological Protection (ICRP) publication 82 [7,8,9,10,11]. Furthermore, because the new standard limits are aimed at long-term application, radionuclides whose half-lives are longer than one year were selected as the regulation target. In particular, Cs-134 and 137, strontium-90, plutonium-238, 239, 240, and 241, and ruthenium-106 were considered to establish new standard limits. Since the measurement of radionuclides other than radioactive cesium require complicated processing, the representative limits for radioactive cesium were established considering the radiation dose from radionuclides other than radioactive cesium [12,13,14,15,16,17]. In other words, the standard limits for radioactive cesium were calculated based on the estimated concentration ratio of each radionuclide in foods [12,17] so that the effective dose from all regulated radionuclides would not exceed 1 mSv/y. Therefore, unless the radioactive cesium in foods exceeds the standard limits (e.g., 100 Bq/kg for general food), the radiation dose from all regulated radionuclides such as strontium-90 does not exceed 1 mSv/y. In fact, the dominant radionuclide in the long term was radioactive cesium, and the contribution of other radionuclides to the radiation dose from foodstuffs was estimated as 12% for age 19 and older when the current limits were derived [17].

Based on the current criteria, many monitoring tests have been mainly conducted by the local government in 17 prefectures located in the eastern part of Japan in accordance with the guideline for the monitoring inspection of radioactive materials in foods [17,18]. Foods containing radioactive materials exceeding the limits are recalled and disposed. Furthermore, the distribution of the food is restricted by type on a prefectural basis (or for smaller areas within a prefecture), if the inspections revealed an increase in the areas where radioactive materials exceed the limits. Thus, rigorous measures are being taken to ensure that foods exceeding the standard limit of radioactive materials are not distributed.

Many monitoring tests have been performed since the provisional regulation values were established in 2011. Approximately 300,000 examinations have been conducted since the current criteria were applied in 2012 [17,19]. The accumulated data are likely to reach 3,000,000 in the near future. The monitoring results are collected and released at the MHLW website, etc. [19,20].

In this study, to evaluate the radiation dose reduction effect by food regulation, the internal exposure dose due to the ingestion of foods after the FDNPP accident was estimated using the accumulated monitoring results. The regulation effect was evaluated by comparing the radiation dose estimated with the results within the standard limits and that estimated with all the monitoring results, which includes the values exceeding the standard limits.

## 2. Materials and Methods

### 2.1. Evaluation Period and Area

**Figure 1:**
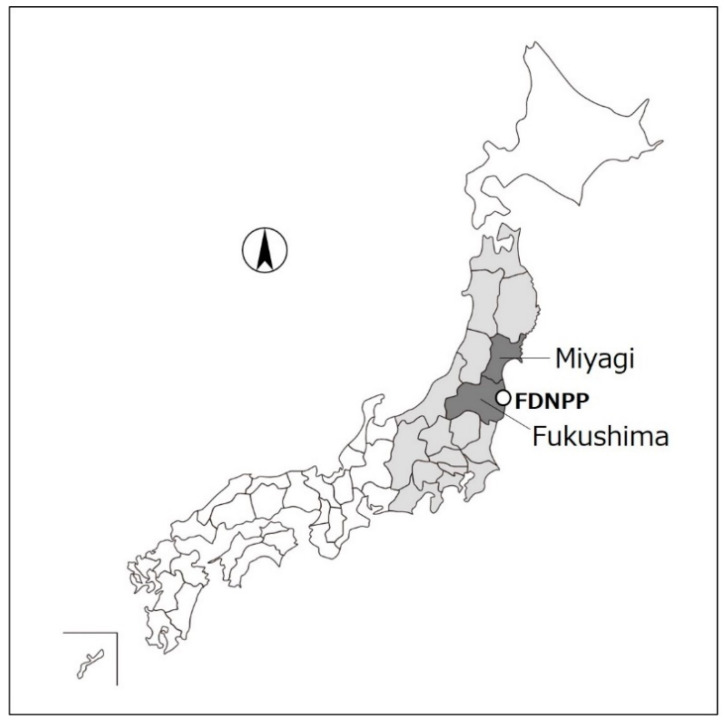
Location of Fukushima and Miyagi Prefectures (dark gray-colored area). The monitoring tests have been performed throughout Japan with a focus on 17 prefectures (dark and light gray-colored area).

In this study, the radiation dose reduction effect was evaluated for FY2012, which is the first year after the current criteria were established and about a year after the FDNPP accident occurred. Additionally, the effect was verified for FY2016 (five years after the FDNPP accident). In addition to the dose estimation with the monitoring results throughout Japan, dose estimation was performed for certain areas that were assumed to be affected heavily (i.e., Fukushima and Miyagi Prefectures) because radionuclides were released in a northwestward direction in the accident (Figure 1) [21].

### 2.2. Data Preparation

#### 2.2.1. Results of the Monitoring Tests

Monthly reports of monitoring results were downloaded from the MHLW website [19]. In each result, the radioactivity concentration of cesium (Bq/kg) is presented with the purchase or sampling day, rough food categories (drinking water, milk/infant food, agricultural product, animal product, fishery product, wild animal meat, and other foods), respective food item names (3119 and 1729 items in FY2012 and FY2016, respectively), production area etc. First, the number and percentage of monitoring results according to the radioactivity concentration in each rough food category were analyzed.

In this study, the total radioactivity concentration of Cs-134 and Cs-137 was used as the radioactivity concentration for radiation exposure dose calculation. The downloaded results in which the total radioactivity concentration of radioactive cesium, purchase or sampling day could not be discriminated were eliminated, and a mixed format of purchase or sampling day was standardized for random sampling as a data cleaning method. The monitoring results were classified for every fiscal year based on the purchase or sampling day. The database was composed with cleaned-up monitoring results.

The radioactivity concentration of brown rice was reduced to a quarter based on a previous report [14,22] to simulate the concentration in the state of ‘ready to eat’ as presented by the CAC [9,23]. Similarly, for a leaf (excluding tea leaves), which supposed to be consumed after extraction as an infusion, the radioactivity concentration was regarded as one-fiftieth based on a previous report [24]. On the other hand, because preparing infusions as measurement samples for green tea leaves is established in the testing method (i.e., as a general rule, the monitoring test result is indicated as the value for the infused liquid) [25], concentration adjustment to simulate the condition in drinking was not performed for green tea leaves. Furthermore, because powdered beverages such as powdered tea is not extracted, not itself consumed and is consumed in a wide variety of ways (i.e., dissolved in water, mixed with other foods, sprinkled on other foods), we did not reduce the concentration. Similarly, in monitoring tests, the standard limit is applied to powdered beverages as it is [26].

#### 2.2.2. Food Intake

The average value of food intake in the results of 2012 National Health and Nutrition Survey Japan was used [27]. The survey data present food intake (g/day) classified into 98 smaller groups of foodstuffs [28]. In our study, the average value of intake for adult men and women (over 20 years old) was used. Furthermore, the intake of drinking water, which is not included in National Health and Nutrition Survey Japan, was set as 2 L/day in the same way as the assumption taken when the new standard limits were derived [12]. Therefore, in all, 99 types of foodstuff were taken into account in food intake.

The food item names in the monitoring test results (3119 and 1729 items in FY2012 and FY2016, respectively) were classified into the 99 types of foodstuff in the food intake data. For instance, ‘broccoli’ in the monitoring results was assigned to ‘Other green and yellow vegetables’ (small classification number 29) in the food intake data. Unnamed sample (e.g., ‘frozen food’) and processed food (e.g., curry) whose main foodstuff could not be identified were eliminated because we could not classify them. Processed food whose main foodstuff could be identified was classified under the main foodstuff. For example, ‘croquette’ was classified as ‘Potatoes and Potato products’.

#### 2.2.3. Dose Coefficient

The dose conversion factor of ingestion intake for an adult as presented in ICRP publication 72 [29] was used to convert the consumed radioactivity (Bq) to internal exposure dose (Sv). The dose coefficients of Cs-134 and Cs-137 for an adult are 1.9 × 10^−8^ and 1.3 × 10^−8^, respectively. The half-lives of Cs-134 and Cs-137 are 2.06 year and 30.2 year, respectively [30]. The weighted averages of Cs-134 and Cs-137 by their half-lives in one year and five years after the FDNPP accident were calculated and used for dose calculation. The weighted average dose coefficients for FY2012 and FY2016 used in this study are listed in Table 2.

### 2.3. Data Acquisition and Dose Calculation

**Figure 2:**
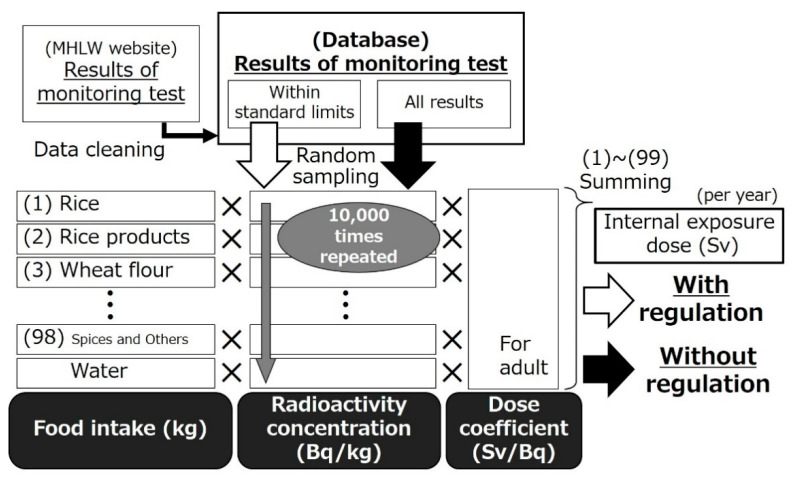
Schema of data acquisition and dose calculation. Random sampling was repeated from the monitoring results within the standard limits or for all results. The radiation exposure dose of virtual 10,000 persons depends on the presence or absence of food regulations was calculated as the product of the food intake, sampled radioactivity concentration in each food and dose coefficient.

The schema of data acquisition and dose calculation is shown in Figure 2. Results of the monitoring test were sampled at random using Rnd function of Microsoft Access 2019 (Microsoft Japan Co., Ltd., Tokyo, Japan) for each food classification. Random sampling was individually performed for the results within the standard limits and all results. The radiation exposure dose when the food was controlled under the current criteria was estimated using the result within the standard limits based on the assumption that food exceeding the limits is not consumed. In contrast, all monitoring data were used in the calculations when shipping restrictions were not taken into account. The method in which the radiation dose is estimated with the monitoring result within the criteria was also adopted when the new standard limit was established by MHLW [12,31]. This methodology is adopted in this study.

Annual radiation exposure dose (committed effective dose) was calculated as the product of the food intake, radioactivity concentration in each food and dose coefficient:

(1) $$\mathrm{Committed}\;\mathrm{effective}\;\mathrm{dose}\;\left(\mathrm{mSv}/y\right)=365.24\cdot 10^{-3}\cdot DCF\sum_{i=1}^{99}I_{i}\cdot C_{i}$$

where *DCF* denotes the dose coefficient (Sv/Bq); *I_i_* denotes the food intake (g/day) in each classification; and *C_i_* denotes the radioactivity concentration of cesium (Bq/kg) sampled randomly corresponding to each food classification.

Random sampling was repeated 10,000 times for each setting. The radiation exposure dose of 10,000 virtual persons was calculated. For the monitoring sample in which radioactive cesium was not detected (ND), the radioactivity concentration was given based on the ratio of the ND samples in each category to calculate the committed effective dose by referring to literature [12,14,32]. When the ND ratio was less than 60% in a category, the radioactivity concentration was set to the value of limit of detection (LOD). When the ND ratio was greater than or equal to 60% and less than 80%, the radioactivity concentration was set to a half of LOD. When the ratio of ND was greater than or equal to 80%, the radioactivity concentration was set to a quarter of LOD. In addition, the radioactivity concentration of food groups that were not tested at all was treated as 0 Bq/kg.

Further, note that setting the LOD equal to or below 1/5 of the standard limit is established in the testing methods for measurement by a germanium semiconductor (HPGe) detector [25]. Furthermore, in the screening method for general foods by a NaI (Tl) scintillation spectrometer, the LOD is equal to or below 25 Bq/kg [33].

## 3. Results

### 3.1. Characteristics of the Monitoring Results

The number and percentage of monitoring results according to radioactivity concentration in each food category in FY2012 and FY2016 are listed in Table 3.

In FY2012, some samples in the categories of drinking water, agricultural product, animal product, fishery product, wild animal meat, and other food exceeded the standard limit. Especially high radioactivity was detected in many samples under the wild animal meat category. Meanwhile, no samples under the milk or infant food category exceeded the standard limit.

In FY2016, the samples exceeding the standard limit decreased considerably, and no sample exceeded the standard limit for the drinking water, milk, or infant food and animal product categories. However, some samples from the wild animal meat and agricultural product categories still showed high radioactivity.

In addition, the breakdown of high radioactivity concentration samples in the FY2012 monitoring results are listed in Table 4. In the table, the top 10 radioactivity concentrations in each percentile (median, 95th and 99th percentiles) in each food classification are indicated along with the food classification. The radioactivity concentration of the ND sample was set to LOD to analyze the monitoring results. A high radioactivity concentration in the median corresponded to other animal meats, including wild boar. Radioactivity in classification for categories excluding other animal meats was similar, i.e., the LOD level. High radioactivity concentrations in the 95th percentile were observed for other animal meats, other poultries (e.g., copper pheasant), mushroom (e.g., log shiitake mushroom, wild mushroom) and other fishes such as demersal fish, freshwater fish. High radioactivity concentrations in the 99th percentile were observed for other beverages, other animal meats, other poultries, mushroom, and other fishes. Other green and yellow vegetables (the 7th highest radioactivity concentration) include wild vegetables.

### 3.2. Estimated Radiation Dose Throughout Japan

**Figure 3:**
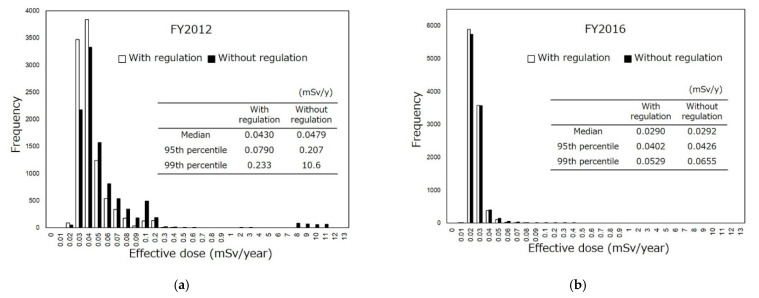
Radiation exposure doses in FY2012 (**a**) and FY2016 (**b**) throughout Japan estimated using 267,717 and 296,708 samples, respectively. Results of unnamed samples and processed foods whose main foodstuffs could not be identified were eliminated from the random sampling.

The distribution of the estimated radiation dose throughout Japan is shown in Figure 3. In the estimation with all test results (assumed not to be regulated: without regulation), the median, 95th percentile and 99th percentile of the committed effective dose in FY2012 were 0.0479, 0.207, and 10.6 mSv/y, respectively. On the other hand, in the estimation with results within the standard limits (assumed to be regulated: with regulation), the median, 95th percentile and 99th percentile of the radiation dose in FY2012 were 0.0430, 0.0790, and 0.233 mSv/y, respectively. In FY2016, the median, 95th and 99th percentile of the radiation exposure dose without regulation were 0.0292, 0.0426, and 0.0655 mSv/y, respectively. With regulation in FY2016, the median, 95th and 99th percentile of the radiation exposure dose were 0.0290, 0.0402, and 0.0529 mSv/y, respectively.

ICRP defined that the radiation dose of the 95th percentile is that received by a ‘representative person’ [34]. In other words, it is believed unless the radiation dose of the 95th percentile exceeds an adopted criterion (e.g., 1 mSv/y for food regulation), a certain group is protected.

**Figure 4:**
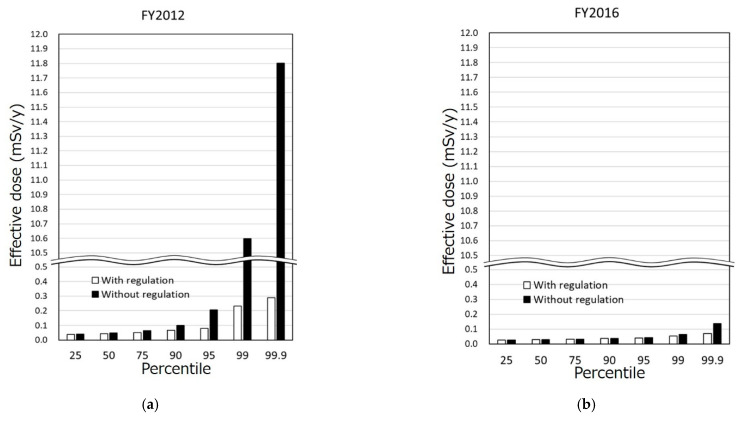
Differences in the radiation dose throughout Japan with or without regulation in each percentile in (**a**) FY2012 and (**b**) FY2016.

The difference in the radiation doses with or without regulation in each percentile are shown in Figure 4. In FY2012 (Figure 4a), the greater the percentile, the larger was the dose reduction effect achieved by adopting the regulation. The radiation doses of the 25th, 50th, 75th, 90th, 95th, 99th, and 99.9th percentile with regulation in FY2012 was 0.93, 0.90, 0.79, 0.66, 0.38, 0.02 and 0.02 times lower than those without regulation. In FY2016 (Figure 4b), dose reduction effects were recognized in the high percentiles; however, the radiation doses were similar in the low percentiles. The radiation dose of 25th, 50th, 75th, 90th, 95th, 99th, and 99.9th percentile with regulation in FY2016 was 0.99, 1.0, 0.99, 0.97, 0.94, 0.81, and 0.51 times those without regulation.

### 3.3. Breakdown of High Radionuclide Intake

The percentile of radionuclide intake without regulation was analyzed for each food classification. The food kind ranking from the 1st to 10th in terms of the radionuclide intake per day in the median, 95th percentile and 99th percentile in virtual 10,000 persons are listed in Table 5. The radionuclide intake was calculated as the product of the sampled monitoring results and food intake.

The medians of the radionuclide intake of rice, water and teas were high. The 95th percentiles of the rice, mushroom, teas, other fruits and other beverages were high. The 99th percentiles of other beverages was extremely high; further, those of mushrooms, other green and yellow vegetables, rice, and other fruits were high.

### 3.4. Estimated Radiation Doses in Fukushima and Miyagi Prefectures

The estimated radiation doses in Fukushima and Miyagi Prefectures are listed in Table 6. The radiation dose of median, 95th and 99th percentile with regulation in FY2012 was 0.77, 0.02, and 0.02 times lower than that without regulation. The radiation dose of median, 95th and 99th percentile with regulation in FY2016 was 0.99, 0.96, and 0.81 times those without regulation.

## 4. Discussion

Almost a decade has passed since the FDNPP accident. Many radionuclide monitoring tests of foods have been conducted continuously. Since these monitoring tests incur some cost, it is important to verify the effect of food regulation in reducing the radiation dose. In this study, the effects of regulations, such as establishing the standard limits, restricting foods that violate the standards, were estimated using the accumulated monitoring results.

In FY2012, many types of samples, except milk or infant foods, exceeded the standard limits as shown in Table 3. In FY2016, the radioactivity concentration and the samples exceeding the standard limits were considerably less than in FY2012. This is because of the decay of radionuclides due to its half-life, weathering effect and measures to reduce the radionuclides in food such as feed management, decontamination of soil and wood and potassium fertilization [35,36,37]. On the other hand, high radioactivity concentration was observed in samples of wild animal meat and agricultural products in FY2016. The samples with as high radioactivity in the categories of wild animal meat include the meat of the wild boar, bear, deer, and wild birds, and those in agricultural products include wild vegetable and mushroom. Since it was difficult to manage feeding or cultivation related to these categories, it is believed that these items contained a high concentration of radioactivity even after several years. In the meanwhile, because there are some wild vegetables cultivated under controlled conditions, restrict distribution are being cancelled for those wild vegetables [38].

In FY2012, the radiation exposure dose with regulation was smaller than that without regulation. Especially, in the high percentiles of radiation dose, the radiation dose with regulation was extremely lower than that without regulation. Therefore, it is thought that the public who would have received a relatively high radiation dose were protected by the adoption of food regulations by the authorities. When provisional regulation values were applied in 2011, the radiation exposure dose was estimated to be 0.139 mSv/y (median) at most from the monitoring results within the provisional regulation values in a similar manner [31]. In our study, the median of internal exposure dose in FY2012 with regulation was estimated to be 0.0430 mSv/y (throughout Japan). The reduction in the radiation dose was likely caused by the decrease in the amount of radionuclides in foods and the adoption of stringent criteria. In FY2016, at high percentiles, the radiation dose with regulation was slightly smaller than that without regulation, while the radiation dose with or without regulation was similar. The impact of radionuclides in foods was considerably small several years after the accident. The radiation exposure dose with regulation in FY2012 and that with and without regulation in FY2016 were significantly small compared to the reference level of 1 mSv/y, which was adopted as the basis for the current criteria. Hence, the measures of foods regulation in Japan after the accident were effective, and the food safety was ensured with regard to radionuclides.

The common samples with high concentrations of radionuclides (95th and 99th percentiles) in the monitoring results (Table 4) were mainly other animal meats, other poultries, mushrooms and other fishes. On the other hand, with regard to the intake of radionuclides (Table 5), common food classification with high radionuclide intake (95th and 99th percentiles) were other beverage, rice, mushroom, teas, other fruits, other fishes, other vegetables, and other animal meats. Other animal meats and other poultries were included as foods with high concentrations of radionuclides (Table 4). However, with regard to the intake of radionuclides (Table 5), other animal meats had low ranking and other poultries were not included as high radionuclide intake. Since the food intake of other animal meats and other poultries were small, it was assumed that the intake of radionuclides from these foods was small. Consequently, it is seen again that the internal radiation exposure dose is determined by not only the radioactive concentration in the food, but also the food intake. Furthermore, although the agricultural product in the monitoring results (Table 3) includes wild vegetable, which often has a high concentration of radionuclides, because the intake volume of wild vegetables was not available, they were classified among other green and yellow vegetables or other vegetables in our study. Therefore, in our dose estimation, the radiation exposure dose arising from consuming wild vegetables might have been underestimated or overestimated. Since wild vegetables are valuable foodstuffs for local residents in Japan [39], in future work, we would like to consider the intake of wild vegetables. Similarly, in terms of food intake, because an average intake was used in this study, biases of individual dietary habits could not be reflected. Furthermore, food intake for processed food was taken into account only for main cooking ingredient. Radionuclides in foodstuff other than the main cooking ingredient in the processed foods were not reflected in the radiation exposure dose. From this viewpoint, the internal exposure dose might have been underestimated to some extent.

The estimated radiation exposure dose (without regulation) with the monitoring results in FY2012 had an extremely high dose (>10 mSv). As shown in Table 5, this high value is mainly attributed to the high radionuclide intake via consumption of other beverages. Specifically, these other beverages were powdered beverages made of plant leaves. The powdered beverage is basically intended to be consumed as a dilute solution. However, as mentioned above, powdered beverage is consumed by itself and is consumed in a wide variety of ways, and we did not adjust the concentration according to the manner of consumption. Therefore, the high radiation dose was perhaps overestimated. Furthermore, because detailed information of the tested samples were not provided in the released monitoring results, there is the possibility that the ‘powdered beverage’ was actually dried and cut leaf (brewed tea is consumed). We tried recalculating the radiation dose under the assumption that the extreme high radioactivity concentration powdered beverage was consumed with one-tenth concentration by dilution. The radiation doses of the 99th and 99.9th percentile without regulation in FY2012 throughout Japan were 1.10 and 1.26 mSv/y, respectively. Furthermore, under the assumption that that powdered beverage was consumed with one-fiftieth concentration by dilution, the radiation doses of the 99th and 99.9th percentile without regulation in FY2012 throughout Japan were 0.285 and 0.518 mSv/y, respectively. Thus, the actual radiation dose of the high percentile may have been smaller. On the other hand, even if the dilution is considered, the high percentile radiation dose without regulation is smaller than that with regulation. Therefore, we regard the food regulation after the FDNPP accident as effective.

Our study was based on the assumption that foodstuffs exceeding the standard limits were not distributed because of the rigorous food management system in Japan. However, some foodstuffs exceeding the standard limit may have been distributed in rare cases. Therefore, our research might have underestimated the radiation exposure dose. However, research using actual distribution of foods (‘a market basket study’) regarding the radionuclides in foods was conducted, and the study showed that the radiation exposure dose due to radioactive cesium was considerably smaller than the reference level of 1 mSv/y [40]. For instance, the radiation dose in 2012 was estimated to be 0.0009~0.0057 mSv/y in market basket study [41]. These results show that the methodology adopted in this study is reasonable. In the meanwhile, the estimated exposure dose in our study was generally large than that by estimated in the market basket study. This is attributed to large LOD in the monitoring tests. Since the monitoring test needs to be conducted conveniently, a larger LOD, which depends on the measurement time, sample volume, etc., is adopted in the monitoring tests than in the market basket study. This is one of the limitations of our research; hence, the internal exposure dose in our study is regarded as overestimated. However, the purpose of our study was to evaluate the effect of food regulation, and because the radiation dose with and without regulation were estimated under identical conditions, we think that the large LOD is not a major problem. Additionally, in the point of view of the overestimation, because monitoring test mainly targets foods which might include high concentration radioactivity, estimated internal exposure dose may be larger than actual radiation dose. Furthermore, radiation dose was estimated based on the assumption that same foods is consumed over a year. The radiation dose for high percentile might have been overestimated, while total diet study is generally conducted in a similar manner.

While the current standard limits were established taking into account radionuclides other than radioactive cesium, such as strontium-90, the internal exposure dose due to radionuclides other than radioactive cesium was not considered in dose estimation in this study. The influence of radionuclides other than radioactive cesium should be included to correctly estimate the radiation dose. However, the dominant radionuclides were Cs-134 and 137, and the concentration of strontium-90 in foods after FDNPP accident was estimated to be within that before the accident [42]. Therefore, we think that the other radionuclides did not affect the estimated dose much.

Despite the limitations of this study, the effect of reduction of the radiation dose by food regulation was verified using the monitoring test results. Since the monitoring test is a meaningful measure of food safety, we would like to continue the investigation on the sequential change of the effect of monitoring tests.

## 5. Conclusions

The radiation dose reduction effect achieved by food regulation was evaluated in this study. The internal exposure dose due to the ingestion of foods after the FDNPP accident was estimated using the accumulated monitoring results effectively. In FY2012, the committed effective dose was considerably small owing to the food regulation. In FY2016, the internal exposure dose with and without the regulations were similar, and the regulation was effective for high percentiles. These doses were significantly smaller than the reference level of 1 mSv/y. Thus, the measures implemented for foods regulation in Japan after FDNPP accident are regarded to have been beneficial for ensuring food safety against radionuclides.

## Figures and Tables

**Table 1:** Current standard limits for radioactive cesium (sum of Cs-134 and Cs-137) in foodstuffs in Japan (applied on 1 April 2012).

Category	Limit (Bq/kg) ^1^
Drinking water	10
Milk	50
General food	100
Infant food	50

^1^ These values consider the contribution of radionuclides other than radioactive cesium, such as strontium-90.

**Table 2:** Used dose coefficients for the sum of radioactivity concentration of Cs-134 and Cs-137.

Dose Coefficient (Sv/Bq)	
FY2012	1.55 × 10^−8^
FY2016	1.40 × 10^−8^

**Table 3:** Number and percentage of monitoring results throughout Japan according to the radioactivity concentration in each rough food category. The percentage is given in parentheses.

	Radioactivity Concentration (Bq/kg)	Drinking Water	Milk/Infant Food	Agricultural Product	Animal Product	Fishery Product	Wild Animal Meat	Other Food
FY2012 ^1^	ND	1197 (70.9%)	5053 (96.1%)	39,732 (78.9%)	177,382 (99.7%)	11,321 (52.9%)	218 (15.9%)	9025 (89.7%)
	0–10	478 (28.3%)	204 (3.9%)	4198 (8.3%)	108 (0.1%)	3272 (15.3%)	62 (4.5%)	371 (3.7%)
	10–25	13 (0.8%)	1 (0.02%)	2665 (5.3%)	284 (0.2%)	2646 (12.4%)	127 (9.2%)	219 (2.2%)
	25–50	0	0	1740 (3.5%)	115 (0.1%)	1899 (8.9%)	197 (14.3%)	188 (1.9%)
	50–100	0	0	1370 (2.7%)	26 (0.01%)	1175 (5.5%)	252 (18.3%)	126 (1.3%)
	100–500	0	0	558 (1.1%)	8 (0.004%)	967 (4.5%)	363 (26.4%)	103 (1.0%)
	500–1000	0	0	74 (0.1%)	0	96 (0.4%)	48 (3.5%)	10 (0.1%)
	1000–5000	0	0	27 (0.1%)	0	17 (0.1%)	74 (5.4%)	10 (0.1%)
	5000–10,000	0	0	1 (0.002%)	0	0	23 (1.7%)	0
	10,000–50,000	0	0	2 (0.004%)	0	0	9 (0.7%)	4 (0.04%)
	50,000–100,000	0	0	0	0	0	2 (0.1%)	0
	Total	1688	5258	50,367	177,923	21,393	1375	10,056
FY2016	ND	507 (99.6%)	3207 (99.8%)	21,451 (83.8%)	238,408 (99.9%)	17,999 (91.0%)	508 (33.4%)	8298 (96.9%)
	0–10	2 (0.4%)	5 (0.2%)	1776 (6.9%)	24 (0.01%)	922 (4.7%)	111 (7.3%)	156 (1.8%)
	10–25	0	0	1533 (6.0%)	89 (0.04)	507 (2.6%)	238 (15.7%)	67 (0.8%)
	25–50	0	0	603 (2.4%)	8 (0.003%)	275 (1.4%)	244 (16.1%)	31 (0.4%)
	50–100	0	0	170 (0.7%)	2 (0.001%)	65 (0.3%)	199 (13.1%)	6 (0.1%)
	100–500	0	0	60 (0.2%)	0	10 (0.1%)	186 (12.2%)	2 (0.02%)
	500–1000	0	0	7 (0.2%)	0	0	19 (1.3%)	0
	1000–5000	0	0	3 (0.01%)	0	0	12 (0.8%)	0
	5000–10,000	0	0	0	0	0	0	0
	10,000–50,000	0	0	0	0	0	2 (0.1%)	0
	50,000–100,000	0	0	0	0	0	0	0
	Total	509	3212	25,603	238,531	19,778	1519	8560

^1^ 31 samples of general foods exceeded 100 Bq/kg in FY2012 but were not treated as violations considering the transitional measure. Note: Monitoring results were classified for each fiscal year based on the purchase or sampling day. Results whose total radioactivity concentration of radioactive cesium, purchase day or sampling day could not be discriminated were eliminated. The double line was used to separate by the standard limits.

**Table 4:** Top 10 classification with high radioactivity in the FY2012 monitoring results. Radioactivity concentrations of these categories in the median, 95th and 99th percentile are indicated along with the food classification name. The radioactivity concentration of ND sample was set to LOD for the analysis.

		The Ranking of Radioactivity Concentration (Bq/kg)									
		1	2	3	4	5	6	7	8	9	10
**Median**	**Radioactivity concentration (Bq/kg)**	**66**	**25–16**								
Small classification ^1^		(64) Other animal meats	Many kinds of foods are included with radioactivity concentration as LOD levels.								
**95th percentile**	**Radioactivity concentration (Bq/kg)**	**2500**	**220**	**170** ^4^	**170** ^5^	**92**	**73.5**	**71**	**62**	**55**	**50**
Small classification ^2^		(64) Other animal meats	(66) Other poultries	(46) Mushrooms	(52) Other fishes	(50) Sea breams and Righteye flounders	(98) Spices and Others	(1) Rice	(49) Salmons and Trouts	(24) Nuts and Seeds	(61) Beefs
**99th Percentile**	**Radioactivity concentration (Bq/kg)**	**17112**	**9000**	**878**	**725**	**491**	**263**	**260**	**251**	**170**	**160**
Small classification ^3^		(91) Other beverages	(64) Other animal meats	(66) Other poultries	(46) Mushrooms	(52) Other fishes	(50) Sea breams and Righteye flounders	(29) Other green and yellow vegetables	(98) Spices and Others	(49) Salmons and Trouts	(43) Other fruits

^1~3^ The number in parentheses represents the small classification number in the National Health and Nutrition Survey Japan. ^4,5^ Radioactivity concentration was the same value.

**Table 5:** Breakdown of high radionuclide intake in FY2012 (without regulation). The top 10 of the 99 food classification in terms of the radionuclide intake per day as calculated by multiplying the sampled monitoring results and food intake are listed. The median, 95th percentile and 99th percentile of the radionuclide intake in virtual 10,000 persons with food intake is represented.

		The Order of Radionuclides Inatake in Each Percentile									
		1	2	3	4	5	6	7	8	9	10
**Median**	**Radionuclides** **intake (Bq/day)**	**0.77**	**0.65**	**0.53**	**0.29**	**0.25**	**0.22**	**0.20**	**0.18**	**0.14**	**0.13**
Food intake (g/day)		328.2	2000	295.6	17.2	63.4	22.7	101.9	39.9	9.0	43.0
Small classification ^1^		(1) Rice	Water	(89) Teas	(46) Mushrooms	(97) Other seasonings	(40) Citrus fruits	(91) Other beverages	(43) Other fruits	(52) Other fishes	(6) Noodles
**95th percentile**	**Radionuclides** **intake (Bq/day)**	**5.9**	**2.8**	**1.9**	**1.7**	**1.6**	**1.5**	**1.2**	**1.0**	**0.93**	**0.90**
Food intake (g/day)		328.2	17.2	295.6	39.9	101.9	9.0	48.3	2000	22.7	0.4
Small classification ^2^		(1) Rice	(46) Mushrooms	(89) Teas	(43) Other fruits	(91) Other beverages	(52) Other fishes	(35) Other vegetables	Water	(40) Citrus fruits	(64) Other animal meats
**99th Percentile**	**Radionuclides** **intake (Bq/day)**	**1861**	**13**	**8.6**	**8.2**	**6.4**	**6.2**	**5.8**	**4.2**	**3.4**	**2.3**
Food intake (g/day)		101.9	17.2	35.9	328.2	39.9	295.6	48.3	9.0	0.4	32.4
Small classification ^3^		(91) Other beverages	(46) Mushrooms	(29) Other green and yellow vegetables	(1) Rice	(43) Other fruits	(89) Teas	(35) Other vegetables	(52) Other fishes	(64) Other animal meats	(32) *Daikon*

^1~3^ The number inside the parentheses represents the small classification number in National Health and Nutrition Survey Japan. Intake of ‘Water’ is not included in the survey.

**Table 6:** Estimated radiation exposure doses in Fukushima and Miyagi area (mSv/y).

		Median			95th Percentile			99th Percentile		
		With Regulation	Without Regulation	(With/Without)	With Regulation	Without Regulation	(With/Without)	With Regulation	Without Regulation	(With/Without)
FY2012	Fukushima, Miyagi ^1^	**0.0552**	**0.0718**	(0.77)	**0.242**	**10.6**	(0.02)	**0.284**	**11.8**	(0.02)
FY2016	Fukushima, Miyagi ^2^	**0.0280**	**0.0282**	(0.99)	**0.0372**	**0.0389**	(0.96)	**0.0445**	**0.0549**	(0.81)

^1,2^ Radiation exposure doses for FY2012 and FY 2016 was estimated using 65,748 and 63,542 samples, respectively. Results of unnamed samples and processed foods whose main contents could not be identified were eliminated from the random sampling.

## Data Availability

Monitoring results used in this study is available at the MHLW website.
